# Clonal integration systemically regulates leaf microstructure of *Bouteloua dactyloides* interconnected ramets to better adapt to different levels of simulated insect herbivory

**DOI:** 10.1093/aobpla/plac062

**Published:** 2022-12-13

**Authors:** Xuxu Chai, Xiaoling Sun, Xinyi Cui, Paul G Johnson, Zhihui Fu

**Affiliations:** College of Horticulture and Landscape Architecture, Tianjin Agricultural University, Tianjin 300392, China; College of Horticulture and Landscape Architecture, Tianjin Agricultural University, Tianjin 300392, China; Department of Plants, Soils and Climate, Utah State University, Logan, UT 84322, USA; College of Horticulture and Landscape Architecture, Tianjin Agricultural University, Tianjin 300392, China; Department of Plants, Soils and Climate, Utah State University, Logan, UT 84322, USA; College of Horticulture and Landscape Architecture, Tianjin Agricultural University, Tianjin 300392, China

**Keywords:** Anatomical structure, *Bouteloua dactyloides*, defense signal, simulated herbivory, vasculature

## Abstract

Stolon connection of clonal plants can translocate resources and signalling molecules between interconnected ramets to enhance resistance. Plants are well known to enhance leaf anatomical structure and vein density to respond to insect herbivory. Herbivory signalling molecules are transferred through vascular system to alert distant undamaged leaves, which is called systemic defence induction. Here, we investigated how clonal integration modulates leaf vasculature and anatomical structure of *Bouteloua dactyloides* ramets to cope with different levels of simulated herbivory. Ramet pairs were subject to six treatments, daughter ramets were exposed to three defoliation levels (0 %, 40 % or 80 % leaf removal) and their stolon connections to mother ramets were either severed or kept intact. Local 40 % defoliation increased vein density and adaxial/abaxial cuticle thickness, decreased leaf width and areolar area of daughter ramets. However, such effects of 80 % defoliation were much smaller. Compared with remote 40 % defoliation, remote 80 % defoliation increased leaf width and areolar area and decreased vein density of interconnected undefoliated mother ramets. Without simulated herbivory, stolon connection negatively affected most leaf microstructural traits of both ramets except from denser veins of mother ramets and more bundle sheath cells of daughter ramets. The negative effect of stolon connection on leaf mechanical structures of daughter ramets was ameliorated in the 40 % defoliation treatment, but not in the 80 % defoliation treatment. Stolon connection increased vein density and decreased areolar area of daughter ramets in the 40 % defoliation treatment. In contrast, stolon connection increased areolar area and decreased bundle sheath cell number of 80 % defoliated daughter ramets. Defoliation signals were transmitted from younger ramets to older ramets to change their leaf biomechanical structure. Clonal integration can adjust leaf microstructure of younger ramets according to the degree of herbivory stress, especially leaf vasculature.

## Teaser

Plants are well known to enhance leaf microstructure to respond to insect herbivory. Herbivory signalling molecules can be transferred through vascular systems to alert distant undamaged leaves. Here, we found that leaf anatomical structure and vasculature of *Bouteloua dactyloides*mother ramets were enhanced when interconnected daughter ramets experienced medium defoliation, but not heavy defoliation. Likewise, clonal integration enhanced leaf microstructure of daughter ramets in the medium defoliation treatment and reduced support for daughter ramets when they were grown in the heavy defoliation treatment. Leaf vasculature was more responsive to remote defoliation and clonal integration than leaf anatomical structure.

## Introduction

In natural habitats, resources and stress factors are heterogeneously distributed. Stolon and rhizome connections between a mother plant and vegetative propagules enable translocation of resources like water, mineral nutrients and assimilates, and plant hormones, pathogens and systemic defence signals (Alpert *et al.* 2002; Stuefer *et al.* 2004; Gómez and Stuefer 2006; Qian *et al.* 2010; Li *et al.* 2011). Physical stresses to the plants like animal trampling and insect herbivory can damage clonal plants, sever stolon/rhizome and defoliate ramets. Mother and daughter ramets respond collectively to the stress experienced by both ramets because of systemic wound signalling. There are three types of wound signals: (i) chemical signals; (ii) physical signals (like hydraulic signals); and (iii) herbivore-induced volatile compounds (Delano-Frier *et al.* 2013). Stuefer *et al.* (2004) suggested that sharing a defence induction signal among ramets is the basis for an efficient early warning system. There is evidence for both phloem and xylem transport of wound signals (Orians 2005). In phloem, an induced resistance signal was mostly reported to transmit from an older to younger ramets due to predominant phloem flow (Gómez and Stuefer 2006; Sui *et al.* 2011). Basipetal translocation of photosynthate and signalling molecules was also detected, especially when older ramets were under stress (Gómez and Stuefer 2006; Qian *et al.* 2010). A hydraulic signal is xylem-borne. Along with water flow in the xylem, chemicals from the wound site can also be drawn into xylem and dispersed away from the wound site, which may even reverse the unidirectional root-to-shoot flow in the xylem (Orians 2005; Delano-Frier *et al.* 2013). Previous reports suggested extensive integration of water resources in clonal plants, which can be translocated both acropetally and basipetally (de Kroon *et al.* 1996; D’Hertefeldt and Jónsdóttir 1999). However, studies linking defence signal transmission to vascular pathways, such as leaf vasculature and stolon/rhizome connection, are still lacking, especially in clonal plants.

Plants have several mechanisms to cope with herbivory, such as resistance against herbivores, tolerance to herbivory or phenological escape (Agrawal 2000). Compensatory growth, one tolerance mechanism, has been largely reported (Strauss and Agrawal 1999; Agrawal 2000; Ferraro and Oesterheld 2002; Hellström *et al.* 2004; Wang *et al.* 2004; Zhao *et al.* 2008; Hamerlynck *et al.* 2016). The compensatory response of clonal plants to partial herbivory damage was also reported to alleviate the detrimental effects of herbivore attack (Gao *et al.* 2008; Zhao *et al.* 2008; Liu *et al.* 2009; Gao *et al.* 2014). However, plasticity of clonal integration in response to simulated herbivory has been generally neglected (Gao *et al.* 2014). Inducible resistance is a form of phenotypic plasticity (Gómez *et al.* 2007). Phenotypic plasticity, which is usually expressed at the level of modular subunits, is a response affected by local environmental condition of individual module, as well as communication and behavioural integration of interconnected modules (de Kroon *et al.* 2005). Gómez *et al.* (2007) found that a stoloniferous plant can systemically activate defence expression after herbivory; this early warning system can bring many benefits, as well as cost if no subsequent herbivory occurs on undamaged ramets. In some cases, this clonal plasticity may even result in no resource support for the damaged ramet, because resource allocation within ramets depends on their relative value (Hellström *et al.* 2006). Therefore, it is critical to assess the cost and benefit of modular plasticity under different stress levels, considering that the degree of tolerance to herbivory is species-specific too (Strauss and Agrawal 1999). For example, how is leaf phenotypic plasticity of interconnected ramets affected by stolon severance, an extreme form of herbivory. Studying modular plasticity can help us better understand how clonal integration modulates clonal plants to respond to different herbivory levels and relate them to plant–environment interactions and fitness consequences.

Induced defence responses after insect herbivory include biomechanical, nutritional and chemical responses (Gómez *et al.* 2008). Unlike chemical defence, mechanical defences, such as leaf anatomical structure and vasculature, are effective under both biotic and abiotic stresses (Read and Stokes 2006). Leaf and cuticle thickness, and lignified vein area are negatively correlated with herbivore density (Peeters 2002). Vein density is positively correlated to resistance to insect herbivory because a higher vein density per unit area forces insects to spend more energy to cut through veins and increases their predation risk (Sack and Scoffoni 2013). Leaf veins in grass leaves are categorized into three types in descending order of resistance: (i) large longitudinal veins, (ii) small longitudinal veins and (iii) transverse veins. Leaf mesophyll tissue exhibits the least resistance (Choong 1996; Méndez-Alonzo *et al.* 2013). The mechanical traits and function of these three types of veins are different. Large longitudinal veins, which originate from the base of the leaf, might involve more construction cost, but are very important in thin and large leaves that are not well protected (Sack and Scoffoni 2013). Small longitudinal veins have similar phloem area throughout the length of the blade, are less tough and do not extend into the sheath region (Ueno *et al.* 2006). Transverse veins possess only one sieve tube (Ueno *et al.* 2006; Sack and Scoffoni 2013). However, to our knowledge, no detailed investigation has examined how clonal integration affects leaf microstructure of clonal ramets after insect herbivory damage, and how phenotypic plasticity helps the whole clone to cope with different levels of insect herbivory stress.

In grassland and lawn ecosystems, insect herbivory and trampling are common stresses that affect growth of clonal plants, which may cause defoliation and sever stolons. *Bouteloua dactyloides*, native to the Great Plain of North America, is widely used for soil and water conservation in Northern China, due to its excellent low-maintenance traits. The reason that *B. dactyloides* is superior to local cold-season species is largely attributed to its strong clonal network (Qian *et al.* 2010; Sun *et al.* 2011; Hao *et al.* 2016). Younger ramets of *B. dactyloides*, the most attractive and vulnerable parts of the clone (Fenner *et al.* 1999), are commonly consumed by locust and other leaf-feeding insects because of the higher nutritional value and lower degree of biomechanical resistance in juvenile leaves (Gómez *et al.* 2008). To examine the effect of clonal integration on leaf microstructure of *B. dactyloides* ramets after insect herbivory damage, we designed a factorial experiment. Apical daughter ramets were grown with three defoliation levels, and their connections to basal mother ramets were either severed or left intact. We addressed the following questions: (i) How does clonal integration affect leaf microstructure of interconnected ramets in the absence of defoliation? (ii) How does local defoliation stress affect leaf microstructure of daughter ramets? (iii) How does remote defoliation stress affect leaf microstructure of interconnected mother ramets? (iv) How does clonal integration affect leaf microstructure of defoliated daughter ramets?

## Materials and Methods

### Experimental design

*Bouteloua dactyloides* ‘Spark’ was selected as the material in this experiment. This C_4_ grass variety has superior shade and drought tolerance, compared to other varieties utilized in Tianjin, China (field observation) (Ren *et al.* 2017). The seeds were provided by Beijing TopGreen Turf & Forage Co., Ltd, and sown in May 2020. On 9 April 2021, 30 ramet pairs of similar size and age, derived from a single clone, were propagated in the glasshouse (18–26 °C) of Tianjin Agricultural University, China (39°16ʹ83″N, 116°98ʹ46″E, 8 m a.s.l.). Each ramet pair consisted of a stolon, a mother ramet (proximal) and a daughter ramet (distal). Each ramet pair was planted in a plastic container (42 × 18 × 13 cm; length × width × height), filled with a mixture of 1:1 vermiculite and peat by volume. A plastic separator was set in the middle of the container to provide two equal separate root compartments. Based on their original size, all ramets were further standardized, with 16 leaves (four tillers) and 6 cm long attached adventitious root. On 10 April 2021, stolon connections on the 15 ramet pairs were randomly selected to sever the stolon connection between the pairs. Stolon connections of the other 15 ramet pairs were kept intact.

To mimic the natural environment where *B. dactyloides* normally propagates, no fertilizer was added. Several studies have reported the negative relationship between nutrient availability and plant tolerance to herbivory, because high nutrients usually lead to reduced root/shoot ratio, which might be associated with reduced tolerance (Strauss and Agrawal 1999; Gao *et al.* 2008). The plants were watered every 2 weeks.

**Figure 1.**
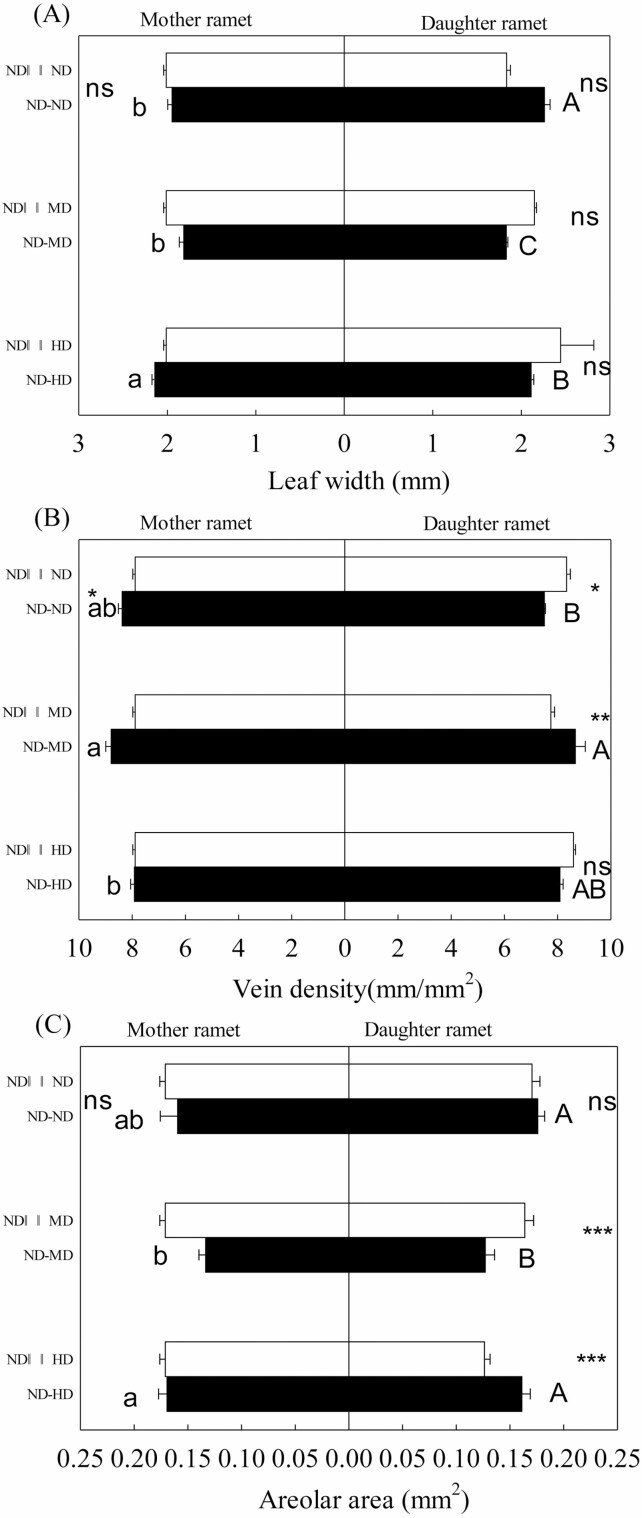
Leaf width (A), vein density (B) and areolar area (C) of mother (left) and daughter (right) ramets of *Bouteloua dactyloides* with the stolon connections either intact (−) or severed (||||). Abbreviation includes ND (no defoliation), MD (medium defoliation) and HD (heavy defoliation). Values are means ± SE. For mother ramets, means with the same lower-case letters between ND–ND, ND–MD and ND–HD are not significantly different (Tukey’s test, *P* < 0.05). For daughter ramets, means with the same capital letters between ND–ND, ND–MD and ND–HD are not significantly different (Tukey’s test, *P <* 0.05). Significance levels within the same defoliation treatment: ^ns^*P* > 0.05; *0.05 ≥ *P* > 0.01; **0.01 ≥ *P* > 0.001; ***P ≤ 0.001.

On 17 April 2021, we subjected 30 daughter ramets to three levels of defoliation treatments: 0 %, 40 % and 80 %, namely no defoliation (ND), medium defoliation (MD) and heavy defoliation (HD), respectively. We removed 16 × 80 % ≈ 13 leaves to represent 80 % defoliation, and 16 × 40 % ≈ 6 leaves to represent 40 % defoliation. Defoliation was conducted on fully expanded leaves and developing leaves were avoided. Stolon connections were kept intact or severed. Ramet pairs of *B. dactyloides* were subjected to one of six treatments (Fig. 1; **see****Supporting Information—Fig. S1**):

- ND||||ND, mother and daughter ramets were not defoliated, and stolon severed;
- ND–ND, mother and daughter ramets were not defoliated, and stolon intact;
- ND||||MD, daughter ramet was 40 % defoliated, mother ramet was not and stolon severed;
- ND–MD, daughter ramet was 40 % defoliated, mother ramet was not and stolon intact;
- ND||||HD, daughter ramet was 80 % defoliated, mother ramet was not and stolon severed;
- ND–HD, daughter ramet was 80 % defoliated, mother ramet was not and stolon intact.

Each treatment had five replicates. The first fully expanded leaf of each ramet was collected as leaf sample for leaf microstructure observation on 13 May 2021. No new ramet was derived on either of the two ramets during this period. The same leaf was used for both leaf vasculature and anatomical structure observation.

### Measurements

**Figure 2.**
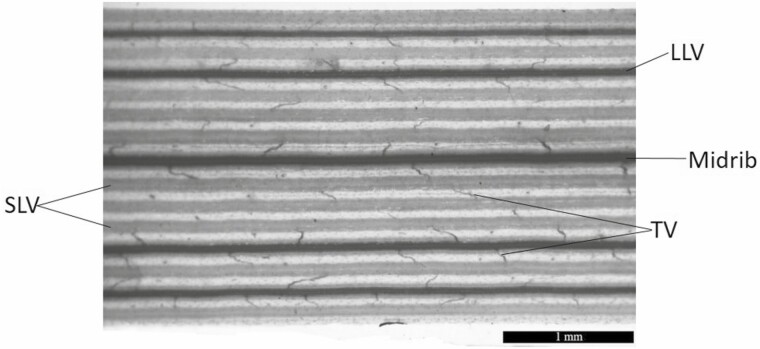
Leaf vasculature of *Bouteloua dactyloides*. LLV, large longitudinal vein; SLV, small longitudinal vein; TV, transverse vein; Midrib is counted as large longitudinal vein.

Leaf vasculature was studied according to Sun *et al.* (2020). The middle portion of the leaf (4–6 cm long) was cleaned with running tap water and dried. Nail varnish was applied to the adaxial epidermis; 10 min later, the film was peeled off from the leaf surface to remove the hairs on the leaf surface. This was repeated on the abaxial side. Leaf segments were then placed in 5 % NaOH for 2 days. Safranin O (1 %) was used to stain leaf segments for 1–2 min, then they were rinsed with distilled water and a graded series of ethanol solutions (30 % → 50 % → 70 % → 80 % → 95 %) in subsequence, each for 5–6 min. Finally, leaf segments were kept in distilled water for 24 h for observation. Leaf vasculature was observed and photographed with Leica S8 APO (Leica, Wetzlar, Germany). Leaf vasculature measurements were made under 10× magnification. Leaf width, distance between small longitudinal veins, distance between transverse veins and densities of the three types of veins were measured by using ImageJ software (National Institutes of Health, USA). Because transverse veins are usually curved (Fig. 2), distance between transverse veins was calculated by the mean of the minimum and maximum distances between transverse veins (Ueno *et al.* 2006). Density of individual vein is the length of each type of vein per unit area. Areolar area was indicated by multiplying the distance between small longitudinal veins by the distance between transverse veins. Fractions of three types of veins were determined by the ratio of each type of vein length to total vein length, respectively. Five different positions were selected for measurement for each leaf segment, totally 25 measurements for each treatment.

**Figure 3.**
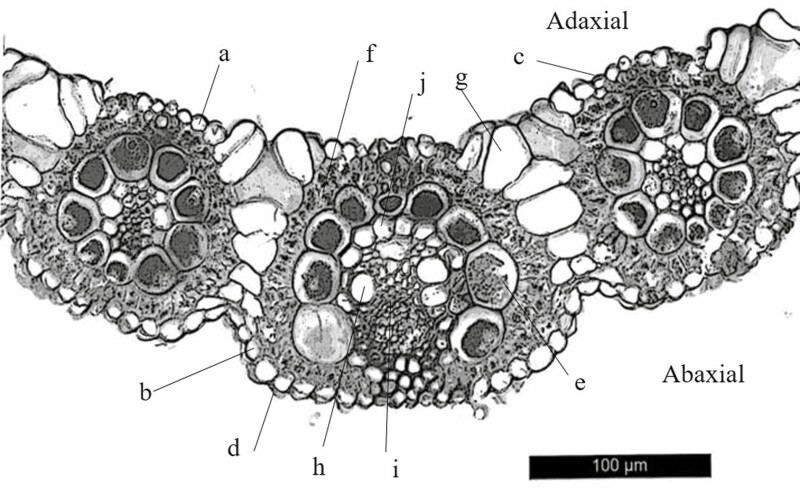
An illustration of anatomical structure of *Bouteloua dactyloides* leaf cross-section. Adaxial epidermal cell (a), abaxial epidermal cell (b), adaxial cuticle (c), abaxial cuticle (d), bundle sheath cell (e), mesophyll cell (f), motor cell (g), xylem (h), phloem (i) and mestome sheath cell (j).

Leaf anatomical structure was measured according to a method of Hao *et al.* (2016). Leaf segments (1 cm long) of newly developed leaves were cut from the middle portion of the lamina, then were fixed in a solution containing formalin, acetic acid and 50 % ethanol (v:v:v = 10:3:87). The fixed segments were then dehydrated in a graded series of ethanol solution, and embedded in Spurr’s resin. The transverse section of the leaf (7–8 μm) was cut with a knife on a microtome, Leica RM2235 (Leica, Wetzlar, Germany), then stained with 1 % safranin O and 2 % fast green. Light microscopy was carried out with Leica DM4000B LED (Leica, Wetzlar, Germany), and a digital camera, Leica DFC450 (Leica, Wetzlar, Germany), was used to take light micrographs. Leaf anatomical measurements were made at 100×. Leaf thickness, midrib diameter, adaxial/abaxial epidermal cell size, adaxial/abaxial cuticle thickness were obtained using LAS AF Lite software (Fig. 3; **see****Supporting Information—Fig. S2**). Like other C_4_ plants, there are two bundle sheath layers in *B. dactyloides* leaves, an inner sheath (mestome sheath) without chloroplasts and an outer sheath (bundle sheath) with chloroplast (Fig. 3). Only bundle sheath cell number was counted in this experiment, because it is closely related to the ability of metabolic transfer to ensure efficient operation of C_4_ photosynthesis between mesophyll cells and bundle sheath cells (Soares-Cordeiro *et al.* 2009). Five different positions were measured in each segment. Investment in leaf venation was calculated by the ratio of midrib diameter to the maximum leaf thickness.

### Data analysis

A paired *t*-test was employed to compare microstructural traits of daughter and mother ramets under ND–ND to illustrate the effect of ramet age on leaf mechanical structure. A two-sample *t*-test was conducted to investigate whether clonal integration affects leaf microstructure of mother ramets by comparing leaf mechanical traits of mother ramets under ND–ND and ND||||ND treatments. One-way ANOVA was used to test the effect of remote defoliation on leaf microstructure of mother ramets by comparing leaf mechanical traits of integrated mother ramets under aforementioned three treatments: ND–ND, ND–MD and ND–HD. Two-way ANOVA was used to test the effects of clonal integration, local defoliation and their interaction on leaf microstructure of daughter ramets. *Post hoc* tests (Tukey’s HSD) were applied to compare leaf mechanical traits of integrated daughter ramets under different levels of clipping treatments (ND–ND, ND–MD and ND–HD) to evaluate the effect of local defoliation. *Post hoc* tests (Simple effect) were carried out to compare the difference between treatments with intact and severed stolon connection at each defoliation level, the right ND parts of ND||||ND and ND–ND, the MD parts of ND||||MD and ND–MD, and the HD parts of ND||||HD and ND–HD, to evaluate the effect of clonal integration on daughter ramets at different defoliation levels. Differences between defoliation levels or severing levels were deemed significant at *P* < 0.05. All statistical analyses were performed with SPSS 20.0 (SPSS, Chicago, IL, USA). The regression equations were generated with Sigmaplot 12.5 software (Systat Software, Inc., USA).

## Results

### Effects of clonal integration in the absence of defoliation

**Figure 4.**
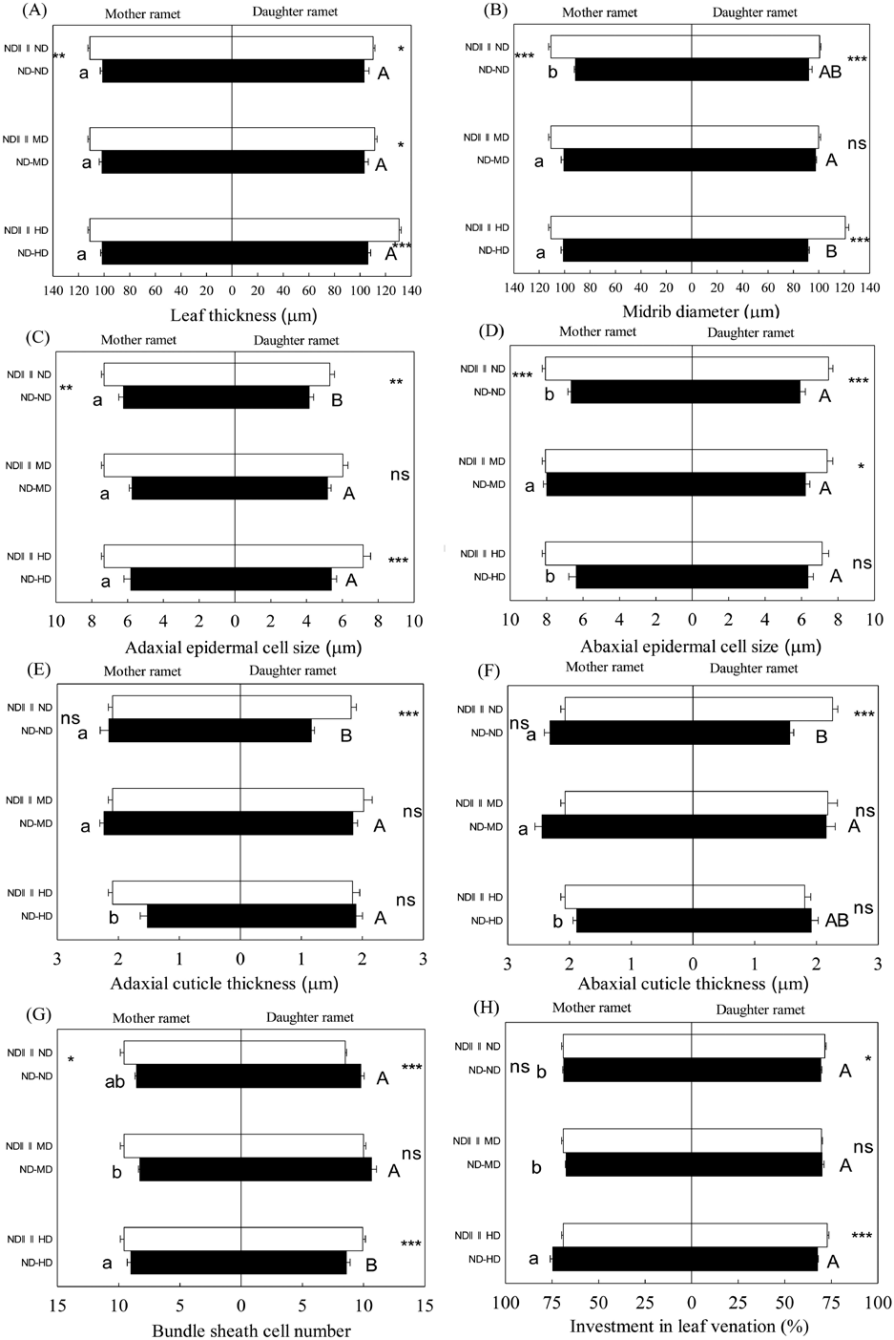
Leaf thickness (A), midrib diameter (B), adaxial epidermal cell size (C), abaxial epidermal cell size (D), adaxial cuticle thickness (E), abaxial cuticle thickness (F), bundle sheath cell number (G), investment in leaf venation (H) of mother (left) and daughter (right) ramets of *Bouteloua dactyloides* with the stolon connections either intact (−) or severed (||||). Abbreviation includes ND (no defoliation), MD (medium defoliation) and HD (heavy defoliation). Values are means ± SE. For mother ramets, means with the same lower-case letters between ND–ND, ND–MD and ND–HD are not significantly different (Tukey’s test, *P* < 0.05). For daughter ramets, means with the same capital letters between ND–ND, ND–MD and ND–HD are not significantly different (Tukey’s test, *P* < 0.05). Significance levels within the same defoliation treatment: ^ns^*P* > 0.05; *0.05 ≥ *P* > 0.01; **0.01 ≥ *P* > 0.001; ***P ≤ 0.001.

**Figure 5.**
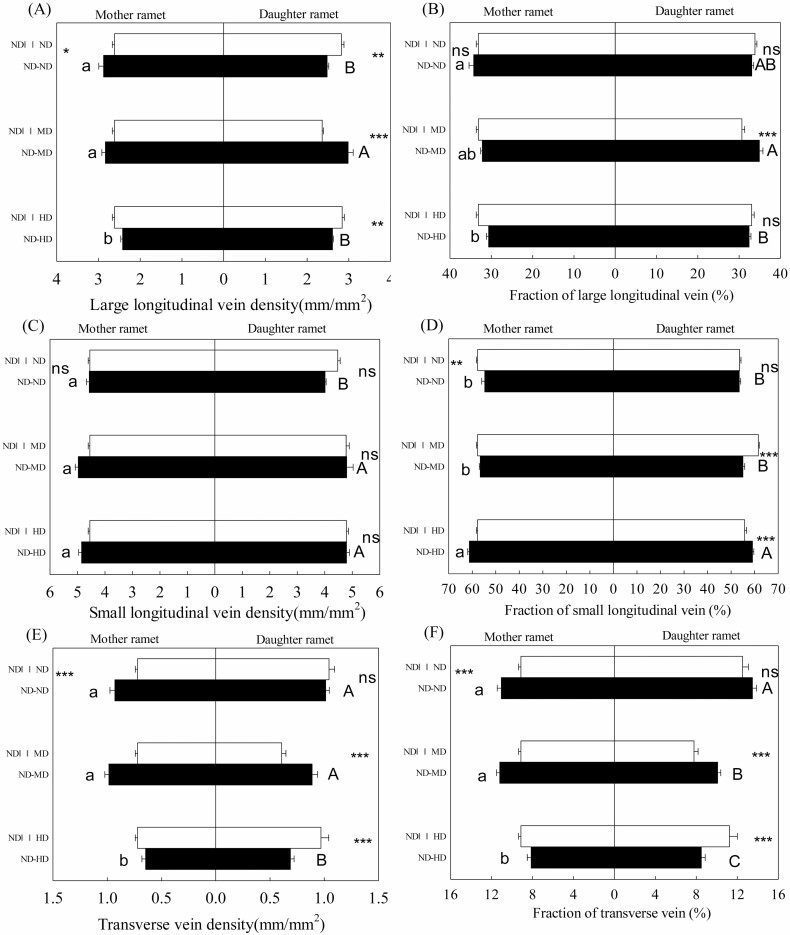
Large longitudinal vein density (A), fraction of large longitudinal vein (B), small longitudinal vein density (C), fraction of small longitudinal vein (D), transverse vein density (E), fraction of transverse vein (F) of mother (left) and daughter (right) ramets of *Bouteloua dactyloides* with the stolon connections either intact (−) or severed (||||). Abbreviation includes ND (no defoliation), MD (medium defoliation) and HD (heavy defoliation). Values are means ± SE. For mother ramets, means with the same lower-case letters between ND–ND, ND–MD and ND–HD are not significantly different (Tukey’s test, *P* < 0.05). For daughter ramets, means with the same capital letters between ND–ND, ND–MD and ND–HD are not significantly different (Tukey’s test, *P* < 0.05). Significance levels within the same defoliation treatment: ^ns^*P* > 0.05; *0.05 ≥ *P* > 0.01; **0.01 ≥ *P* > 0.001; ***P ≤ 0.001.

Mother ramets showed higher degree of leaf biomechanical resistance than interconnected unstressed daughter ramets, with narrower leaves (Fig. 1A; *P* < 0.001), denser veins (Fig. 1B; *P* < 0.001), bigger adaxial/abaxial epidermal cell (Fig. 4C and D; *P* < 0.01) and thicker adaxial/abaxial cuticle (Fig. 4E and F; *P* < 0.001). In the absence of defoliation, stolon connection had positive effects on leaf vasculature of mother ramets. Severing stolons reduced vein density of mother ramets, especially the densities of large longitudinal veins and transverse veins (Figs 1B and 5A, E). In contrast, leaf thickness, midrib diameter, adaxial/abaxial epidermal cell size and bundle sheath cell number of mother ramets were all negatively affected by stolon connection, and severing stolons drastically increased these parameters (Fig. 4A–D and G). Meanwhile, severing stolons increased newly developed leaf numbers and tillering numbers of mother ramets, around 2–3 new leaves and 2–3 new tillers developed in ND||||ND treatment, but only 1–2 new leaves and no new tillers developed in ND–ND treatment. With the exception of small longitudinal vein density, all microstructural parameters of daughter ramets were significantly affected by stolon connection or the effects of interaction between stolon connection and defoliation (Tables 1 and 2). Severing stolons markedly increased vein density, and all anatomical parameters of daughter ramets apart from bundle sheath cell number (Figs 1B and 4). Severing stolons also increased tiller numbers of undefoliated daughter ramets, two new tillers developed in ND||||ND treatment, but none developed in ND–ND treatment.

**Table 1.** Analysis of variance for the effects of clonal integration, defoliation and their interaction on leaf vascular system of *Bouteloua dactyloides* daughter ramets. *F*-values are shown for each variable followed by their respective significance levels. Significance levels: *0.05 ≥ *P* > 0.01; **0.01 ≥ *P* > 0.001; ***P ≤ 0.001. LV, longitudinal vein; TV, transverse vein.

		Integration (*I*)	Defoliation (*D*)	*I* × *D*
Variable	DF	1, 124	2, 124	2, 124
Leaf width (mm)		0.362	2.402	3.574*
Vein density (mm/mm^2^)		0.645	1.844	9.615***
Areolar area (mm^2^)		0.026	6.640**	10.738***
Large LV density (mm/mm^2^)		0.082	0.612	29.785***
Fraction of large LV (%)		3.029	0.614	9.887***
Small LV density (mm/mm^2^)		1.407	7.552**	1.405
Fraction of small LV (%)		3.810	21.986***	29.889***
TV density (mm/mm^2^)		0.071	14.342***	18.633***
Fraction of TV (%)		0.191	31.333***	16.244***

**Table 2.** Analysis of variance for the effects of clonal integration, defoliation and their interaction on leaf anatomical structure of *Bouteloua dactyloides* daughter ramets. *F*-values are shown for each variable followed by their respective significance levels. Significance levels: *0.05 ≥ *P* > 0.01; **0.01 ≥ *P* > 0.001; ***P ≤ 0.001.

		Integration (*I*)	Defoliation (*D*)	*I* × *D*
Variable	DF	1, 81	2, 81	2, 81
Leaf thickness (µm)		46.376***	16.155***	9.052***
Midrib diameter (µm)		93.502***	19.790***	34.738***
Adaxial epidermal cell size (µm)		25.518***	14.454***	1.145
Abaxial epidermal cell size (µm)		22.860***	0.058	0.954
Adaxial cuticle thickness (µm)		9.410**	11.201***	6.751**
Abaxial cuticle thickness (µm)		4.957*	3.885*	7.892***
Bundle sheath cell number		0.823	11.871***	15.710***
Investment in leaf venation (%)		15.353***	0.280	6.517**

### Effects of local defoliation on daughter ramets

**Figure 6.**
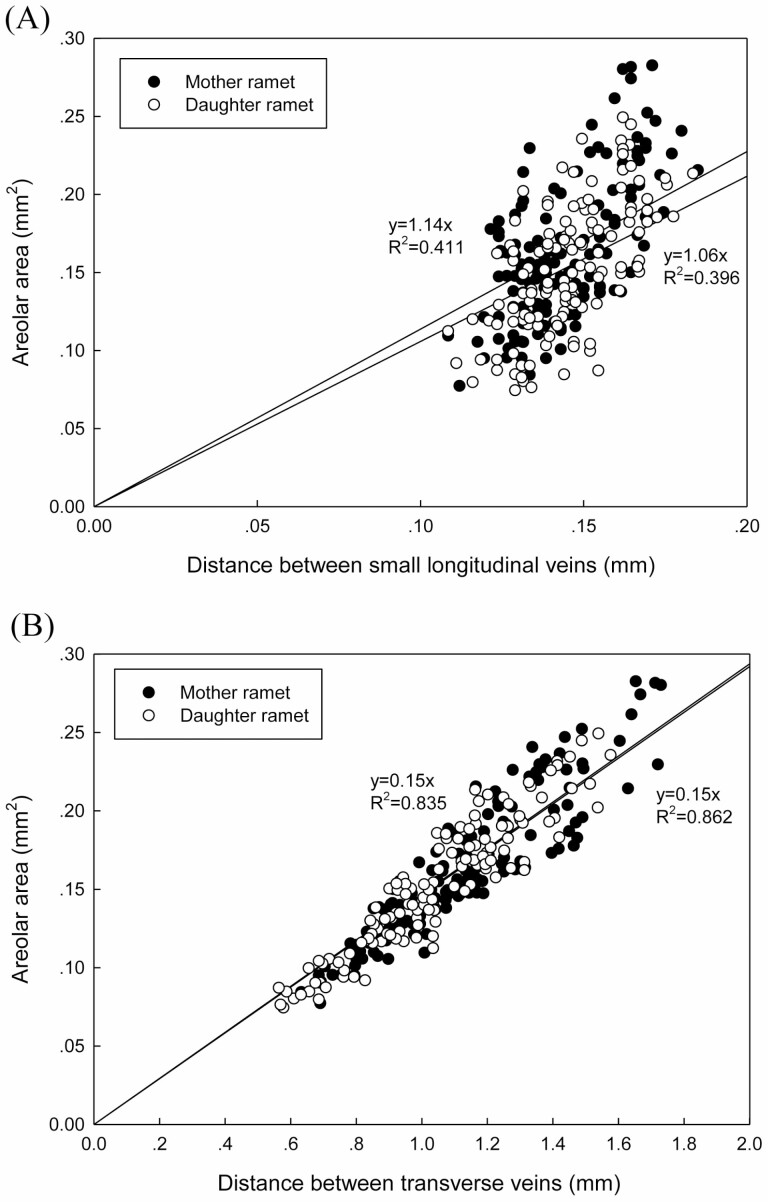
Relationship of areolar area and distance between small longitudinal veins (A), and relationship of areolar area and distance between transverse veins (B).

When daughter ramets were integrated with mother ramets, they developed 3–4 new leaves and 3 new tillers, 7 new leaves and 1 new tiller in MD and HD treatments, respectively. Local defoliation had significant effects on leaf vasculature and anatomical structure of daughter ramets (Tables 1 and 2). Local MD increased vein density, decreased leaf width and areolar area of daughter ramets (Fig. 1A–C); it also increased adaxial epidermal cell size, and adaxial/abaxial cuticle thickness (Fig. 4C, E and F) of daughter ramets. Whereas the effect of local HD tended to be smaller, vein density and areolar area of daughter ramets were not notably altered (Fig. 1B and C), bundle sheath cell number was even decreased, in comparison with that of daughter ramets under the ND treatment (Fig. 4G). However, the density and fraction of small longitudinal veins were greatly increased by local HD (Fig. 5C and D). Under different treatments, fractions of large longitudinal veins, small longitudinal veins and transverse veins ranged from 30.7–34.9 %, 53.5–61.1 % and 7.8–13.5 %, respectively (Fig. 5B, D and F), suggesting greater variation of transverse veins. Areolar areas of both ramets were more closely correlated with distances between transverse veins (mother ramet: *R*^2^ = 0.835; daughter ramet: *R*^2^ = 0.862) than with distances between small longitudinal veins (mother ramet: *R*^2^ = 0.411; daughter ramet: *R*^2^ = 0.396) (Fig. 6).

### Effects of remote defoliation on mother ramets

Mother ramets developed 7–8 new leaves and 4 new tillers in the ND–MD treatment, 2–3 new leaves and 1 new tillers in the ND–HD treatment. Leaf microstructure of mother ramets was pronouncedly affected by remote defoliation stress. Remote MD increased midrib diameter and abaxial epidermal cell size of undefoliated mother ramets (Fig. 4B and D). Compared with remote MD, the effect of remote HD was larger and quite opposite (Figs 1 and 4), remote HD decreased vein density of undefoliated mother ramets, increased leaf width and areolar area (Fig. 1A–C). Remote HD also increased midrib diameter and investment in leaf venation and decreased adaxial/abaxial cuticle thickness of undefoliated mother ramets (Fig. 4B, E, F and H). Fractions of small longitudinal vein of mother ramets were greatly increased by remote HD, at the cost of the other two types of vein fractions (Fig. 5B, D and F).

### Effects of clonal integration on defoliated daughter ramets

After being severed from mother ramets, around 5 and 12 new leaves were developed by daughter ramets in ND||||MD and ND||||HD treatments, respectively, but both of them only developed one new tiller. Under MD, stolon connection had positive effects on leaf vasculature of daughter ramets, severing stolons led to bigger areolar area and reduced vein density, densities and fractions of large longitudinal veins and transverse veins were much smaller after severing (Figs 1B, C and 5A, B, E, F). The negative effect of stolon connections on leaf anatomical traits of unstressed daughter ramets was greatly alleviated when they were grown under MD; only leaf thickness and abaxial epidermal cell size were increased after severing stolons (Fig. 4A and D). However, most microstructural traits of daughter ramets were negatively affected by stolon connection again when grown under HD, though compared to unstressed daughter ramets, the negative effects were much smaller. Apart from adaxial/abaxial cuticle thickness and abaxial epidermal cell size, severing mother–daughter connections increased most anatomical traits and decreased areolar area of daughter ramets under HD (Figs 1C and 4). Fractions of small longitudinal veins were greatly reduced and densities of large longitudinal veins and transverse veins of daughter ramets under HD were increased after severing (Fig. 5A, D and E).

## Discussion

### Integration effects on mother and daughter ramets without simulated insect herbivory

Without simulated herbivory, clonal integration strengthened the vascular system of mother ramets by increasing vein density and reducing leaf number, leaf thickness, midrib diameter and adaxial/abaxial epidermal cell size. High vein density normally corresponds to smaller epidermal cell size, and is very important in thinner leaves that are not well protected (Sack and Scoffoni 2013). Thinner leaves and high vein density increase contact between vascular and photosynthetic tissue and reduced vein diameter allows bending so that leaf centre can face towards sunlight. High vein density also enables greater phloem transport efficiency, and equalizes water potential across the leaf. Combined with the fact that more new leaves and tillers were developed by mother ramets after severing stolons, it is speculated that these traits may help mother ramets to efficiently transport photo-assimilate to interconnected daughter ramets.

In contrast, clonal integration had negative effects on leaf venation of unstressed daughter ramets, and most leaf anatomical traits were negatively affected by clonal integration too, except from bundle sheath cell number. Different from other mechanical structures, the main function of bundle sheath cells is metabolite transfer associated with photosynthesis (Soares-Cordeiro *et al.* 2009). Clonal integration increased bundle sheath cell number of unstressed daughter ramets for more efficient metabolic translocation, which is congruent with the predominant phloem flow of photosynthate in clonal plants. The possible explanation for less investment in leaf microstructure of daughter ramets and anatomical structure of mother ramets in unstressed conditions is that *B. dactyloides* is a ‘guerilla’ type clonal species (Qian *et al.* 2010); it may invest more biomass for vegetative propagation and exploration of new patches in natural environment, rather than construction of leaf biomechanical traits, since these mechanical traits may have downstream effects on plant productivity (Read and Stokes 2006). For this reason, more new tillers were developed by both ramets after severing stolons.

### Local defoliation effects

Local MD significantly increased vein density, decreased areolar area of daughter ramets, denser veins and smaller areolar area can provide an advantage against insect herbivory, since insects need to spend more energy to cut through those structures (Read and Stroke 2006; Sack and Scoffoni 2013). Local MD also substantially increased adaxial epidermal cell size and adaxial/abaxial cuticle thickness of daughter ramets. A thicker cuticle can reduce herbivory by increasing leaf stiffness and strength (Gutschick 1999; Read and Stokes 2006). A higher degree of leaf biomechanical resistance may lead to reduced palatability of daughter ramets with MD treatment, and thereby increase protection of vulnerable younger ramets. Local HD reduced bundle sheath cell number of daughter ramets. For C_4_ plants like *B. dactyloides*, their leaves are characterized by Kranz-type anatomy, decarboxylation and refixation of CO_2_, all occur in the bundle sheath cell; therefore, bundle sheath cell number is closely related to the efficiency of metabolite transfer and C_4_ photosynthesis (Jiang *et al.* 2011; Sack and Scoffoni 2013). Similar results have been reported by Zhao *et al.* (2008), *Leymus chinensis* with heavy clipping treatments (60 %) showed significant lower photosynthetic rate. Here, the effect of local HD on leaf mechanical traits of daughter ramets tended to be smaller, in comparison with local MD. Considering the fact that more new leaves and less new tillers were developed by daughter ramets under HD than under MD, it is speculated that after cost–benefit trade-offs evaluation, daughter ramets ‘realized’ that it was more meaningful to develop more leaves and increase photosynthetic tissue volume, rather than enhancing leaf biomechanical traits, since 80 % leaves were removed already.

### Remote defoliation effects

In contrast to the plastic responses triggered by remote MD, remote HD increased leaf width, decreased vein density and adaxial/abaxial cuticle thickness of the undefoliated mother ramets. Reduced vein density allows for displacement of mesophyll (Sack and Scoffoni 2013). Meanwhile, remote HD increased plant height and stem diameter of mother ramets **[see****Supporting Information—Fig. S3****]**, these responses were consistent with increased leaf width, indicating that remote HD may increase photosynthetic tissue volume of mother ramets to capture more sunlight and produce more photosynthate. The plant cuticle is a hydrophobic membrane, which generally consists of inner cutin and outer wax to prevent water loss. A thickened cuticle can also protect against leaf herbivory (Müller 2006; Zhang *et al.* 2012; Han *et al.* 2021). The remote HD signalled the mother ramets to decrease vein density and cuticle thickness, reduce investment on leaf mechanical properties and construction costs to produce wider less expensive leaves, which may facilitate a higher rate of carbon gain (Wright and Cannon 2001; Read and Stokes 2006; Carins Murphy *et al.* 2016). Likewise, due to high herbivory rate, early successional species in a tropical forest produced low-cost, short-lived leaves (Poorter *et al.* 2004). Another possible explanation is that decrease in leaf biomechanical properties of mother ramets may increase attractiveness of older ramets compared to damaged young ramets, and help to spread the risk of attack to confer protection of more valuable young ramets (Gómez *et al.* 2008). Meanwhile, less new leaves and new tillers were developed by mother ramets when connected with daughter ramets under HD than under MD. Taken together, remote MD regulated undefoliated mother ramet to develop more leaves and strengthen leaf mechanical traits, whereas remote HD modulated undefoliated mother ramet to reduce leaf mechanical construction cost, produce less leaves, but with increased leaf width. These contrasted responses demonstrated that *B. dactyloides* may systemically regulate undamaged ramets to respond according to the level of remote herbivory stress.

### Integration effects on defoliated daughter ramets

Many studies have shown that clonal integration may improve compensatory growth of grazed plants (Zhao *et al.* 2008; Liu *et al.* 2009; Gao *et al.* 2014). Our results were consistent with this notion, clonal integration increased vein density and decreased areolar area of daughter ramets under MD to help it to be more resistant to simulated herbivory. The negative effects of clonal integration on leaf mechanical traits of unstressed daughter ramets were ameliorated when daughter ramets were under MD. However, this support was withdrawn when daughter ramets were under HD, areolar area was increased and bundle sheath cell number was greatly reduced by clonal integration. Reduced bundle sheath cell number was associated with reduced ability to transfer metabolites and less support from mother ramets. One possible explanation was that support of a module depends on its relative value; support for an inferior module may cease after severe and prolonged stress because the profit gained from support outweigh the investment (de Kroon *et al.* 2005; Hellström *et al.* 2006). Correspondingly, after severing stolons, more new leaves were developed by daughter ramets in the ND||||HD treatment, suggesting that resource support for daughter ramets in the ND–HD treatment ended or diverted. Another herbaceous plant, *Psammochloa villosa*, which also has a guerilla growth form, invested more energy in the production of new ramets under heavy clipping (90 % shoot removal), rather than in regrowth of grazing ones (Liu *et al.* 2009). Mechanical traits of daughter ramets under HD were not strengthened by clonal integration, neither were those of interconnected mother ramets, indicating that to allocate more resources for intact ramets or for photosynthetic tissue should be a better option for *B. dactyloides* under HD. Therefore, clonal integration may strengthen leaf biomechanical traits of daughter ramets under MD, and reduce support for daughter ramets when they were grown under HD.

### Functions of three types of veins

With the increment of defoliation stress, the fractions of small longitudinal veins increased consistently, for both mother and daughter ramets. It is reported that there is a trade-off between the mechanical properties of leaf tissue and the fraction of tissue invested in support. Plants with high material resistance and density deployed a low investment in the support tissue of midrib (Niinemets *et al.* 2007a, b; Méndez-Alonzo *et al.* 2013). Consistent with these results, daughter ramets under defoliation stress invested relatively less proportion of biomass in large longitudinal veins and more in small longitudinal veins. Similarly, remote defoliation increased the fraction of small longitudinal veins in mother ramets at the cost of the other two types of veins, further confirmed that small longitudinal vein should play an important role in mechanical resistance and protection again herbivore damage in *B. dactyloides*.

The densities of large longitudinal vein and transverse vein of daughter ramets were increased when they were grown under MD, and decreased under HD due to clonal integration. We suspect that these two types of veins are vital for daughter ramets to receive more/less resource subsidy from a mother ramet. Large longitudinal veins serve for transport of nutrients in/out of leaf blade; transverse veins that connect longitudinal veins are responsible for lateral transport of nutrients. Denser transverse veins led to smaller areolar area (Fig. 6B), which not only enable more efficient photosynthate translocation, but also better protection against insect herbivory (Ueno *et al.* 2006; Sack and Scoffoni 2013). Compared to anatomical structure, clonal integration had greater impact on leaf vascular system of both ramets under simulated herbivory stress. It was reported that defence signal induction from a damaged leaf to undamaged leaves is strongly associated with vascular connectivity between these leaves (Jones *et al.* 1993; Orians *et al.* 2000; Orians 2005; Ferrieri *et al.* 2015). Similar to the function of stolons, transport of photo-assimilate, signal molecules and hormones between leaves is largely determined by vascular architecture (Orians 2005). To our knowledge, this is the first study to demonstrate that clonal integration modified leaf vasculature of interconnected ramets to adapt to different levels of simulated herbivory stress.

## Conclusions

In natural grassland and lawn ecosystems, insect and herbivore may exert constant biotic herbivory stress on plant growth, which may cause defoliation and stolon/rhizome severance. Our study showed that induced defence signals can be transmitted from defoliated younger ramets of *B. dactyloides* to older ramets; clonal integration may regulate leaf microstructure of interconnected ramets according to the degree of herbivory stress. However, because insect herbivory is a rather uncertain and complex event, leaf microstructural response could also be related to resource reserves and resource uptake efficiency; signal and resource transmission between *B. dactyloides* ramets may therefore be more complicated than shown in our experiment. We propose that the function of stolons is far more than translocation of resources, but also coordinating the microstructure of different clonal parts, especially the leaf vascular system of interconnected ramets, for better performance of the whole genet. To allow more robust extrapolation, more physiological studies should be combined to explore the deeper mechanism behind stress signal transmission and clonal integration.

## Supporting Information

The following additional information is available in the online version of this article—

**Figure S1.** Schematic diagram of experimental design. Stolon connections of *Bouteloua dactyloides* interconnected ramets were either intact (−) or severed (||||). Abbreviation includes ND (no defoliation), MD (medium defoliation) and HD (heavy defoliation).

**Figure S2.** A full illustration of anatomical structure of *Bouteloua dactyloides* leaf cross-section.

**Figure S3.** Plant height (A) and stem diameter (B) of mother (left) and daughter (right) ramets of *Bouteloua dactyloides* with the stolon connections either intact (−) or severed (||||). Abbreviation includes ND (no defoliation), MD (medium defoliation) and HD (heavy defoliation). Values are means ± SE. For mother ramets, means with the same lower-case letters between ND–ND, ND–MD and ND–HD are not significantly different (Tukey’s test, *P* < 0.05). For daughter ramets, means with the same capital letters between ND–ND, ND–MD and ND–HD are not significantly different (Tukey’s test, *P* < 0.05). Significance levels within the same defoliation treatment: ^ns^*P* > 0.05; **0.01 ≥ *P* > 0.001.

plac062_suppl_Supplementary_Figures

## Data Availability

The data that support the findings of this study are incorporated into the article and its **Supporting Information**. Further inquiries can be directed to the corresponding author.

## Notes

**Form & Function. Chief Editor**: Kate McCulloh
