# Supplementary material for: Clonal integration systemically regulates leaf microstructure of Bouteloua dactyloides interconnected ramets to better adapt to different levels of simulated insect herbivory
Source: AoB Plants. 2022 Dec 13;15(2):plac062. doi: 10.1093/aobpla/plac062 (PMC9948802; doi:10.1093/aobpla/plac062)
Supplement: plac062_suppl_Supplementary_Figures [file plac062_suppl_supplementary_figures.docx]

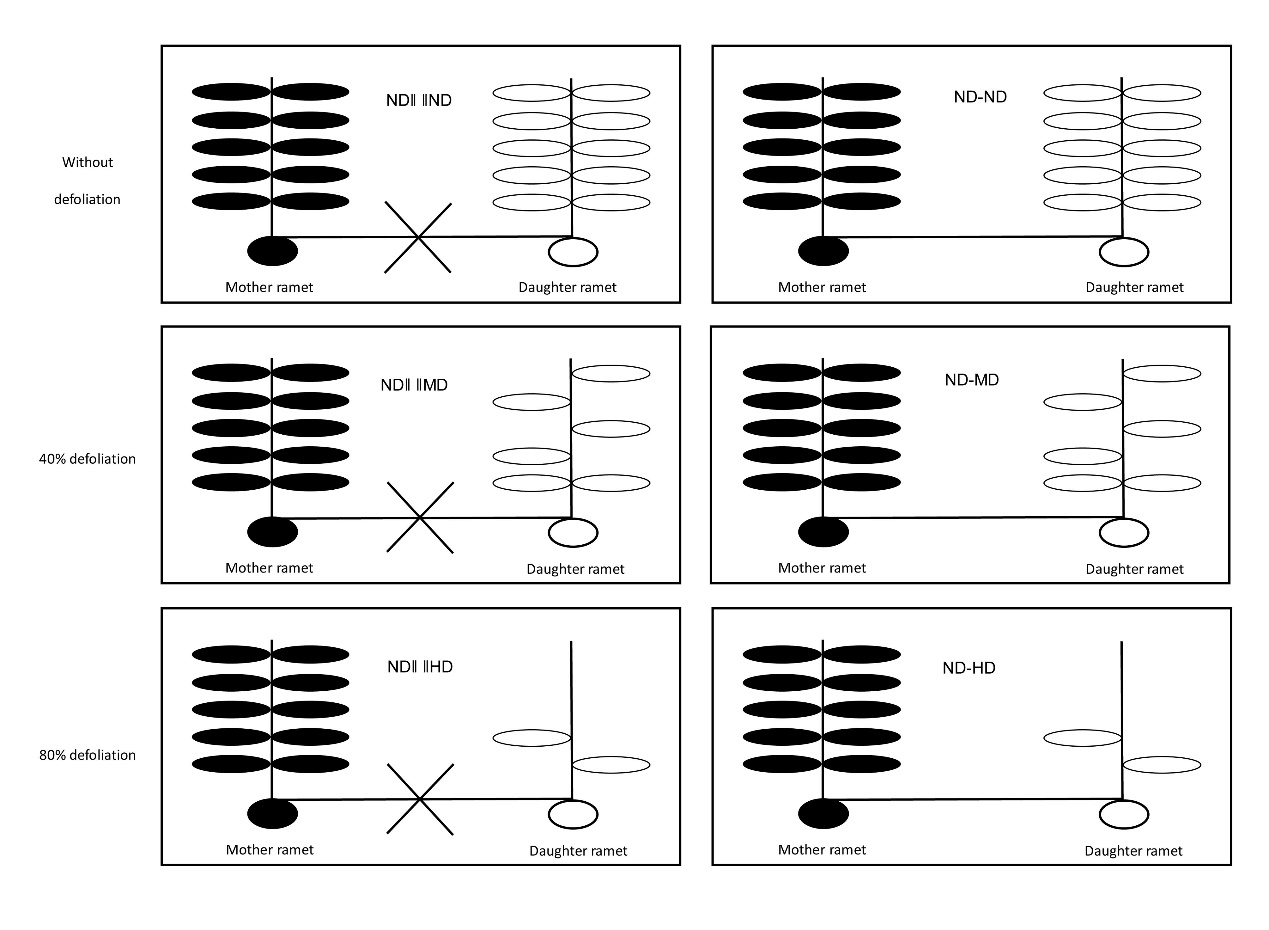


**Figure S1.** Schematic diagram of experimental design. Stolon connections of *Bouteloua dactyloides* interconnected ramets were either intact (-) or severed (‖‖). Abbreviation includes ND (no defoliation), MD (medium defoliation), and HD (heavy defoliation).


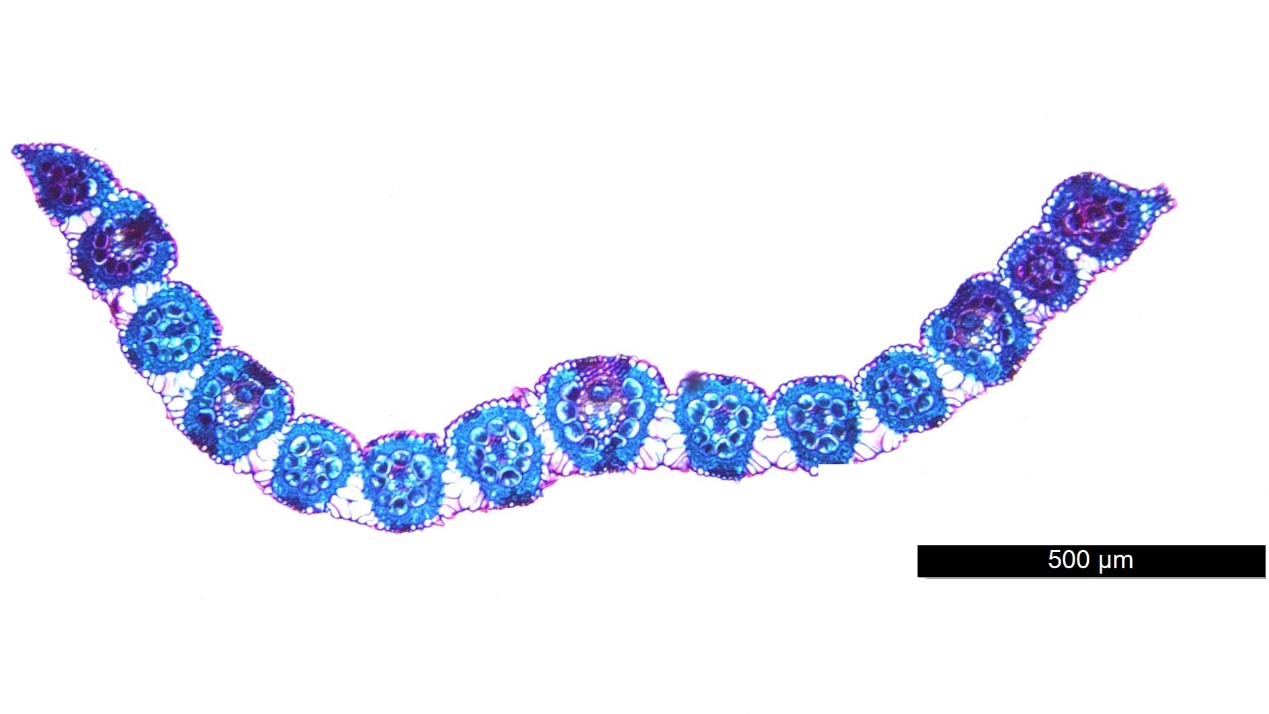


**Figure S2.** A full illustration of anatomical structure of *Bouteloua dactyloides* leaf cross-section.


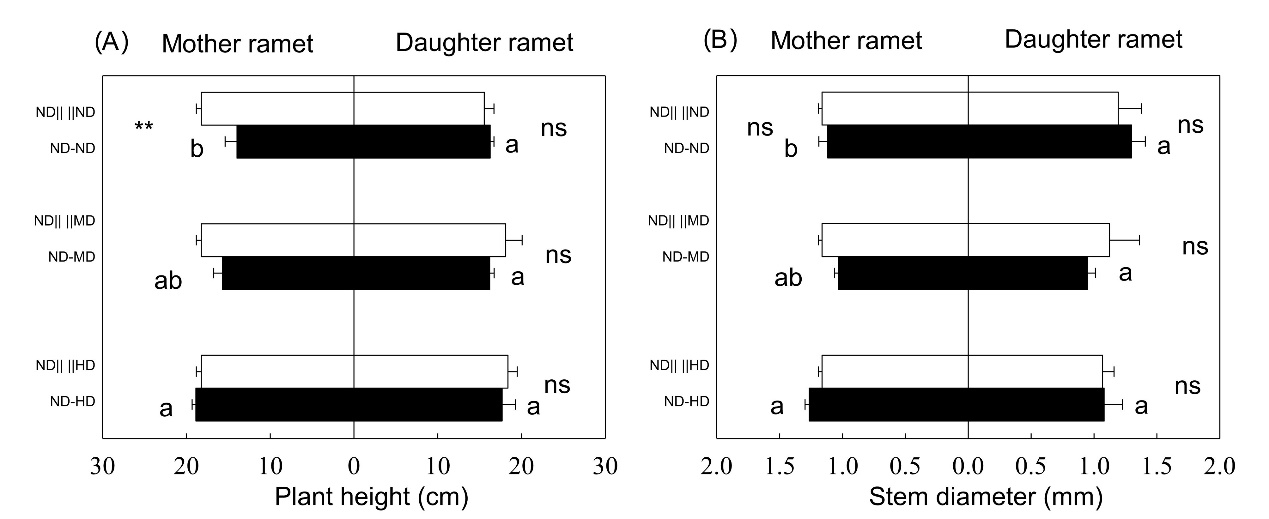


**Figure S3.** Plant height (A) and stem diameter (B) of of mother (left) and daughter (right) ramets of *Bouteloua dactyloides* with the stolon connections either intact (-) or severed (‖‖). Abbreviation includes ND (no defoliation), MD (medium defoliation), and HD (heavy defoliation). Values are means±s.e. For mother ramets, means with the same lower-case letters between ND-ND, ND-MD and ND-HD are not significantly different (Tukey’s test, *P*<0.05). For daughter ramets, means with the same capital letters between ND-ND, ND-MD and ND-HD are not significantly different (Tukey’s test, *P*<0.05). Significance levels within the same defoliation treatment: ^ns^, *P* > 0.05; ^*^, 0.05 ≥ *P* > 0.01; ^**^, 0.01 ≥ *P* > 0.001; ^***^, *P*≤ 0.001
